# Supplementary material for: Resolving the full spectrum of human genome variation using Linked-Reads
Source: Genome Res. 2019 Apr;29(4):635–45. doi: 10.1101/gr.234443.118 (PMC6442396; doi:10.1101/gr.234443.118)
Supplement: Supplemental Material [file supp_gr.234443.118_Supplemental_longranger-2.2.zip › longranger-2.2.tar.bz2!/lr2.2-public/lib/stan/cmdstan/stan/doc/api/html/pareto_8hpp_source.html]

Stan: src/stan/prob/distributions/univariate/continuous/pareto.hpp Source File


|  |  |
| --- | --- |
|  | Stan  2.5.0  probability, sampling & optimization |


- Main Page
- Related Pages
- Namespaces
- Classes
- Files

- File List
- File Members

All Classes Namespaces Files Functions Variables Typedefs Enumerations Enumerator Friends Macros Pages

- src
- stan
- prob
- distributions
- univariate
- continuous

pareto.hpp

Go to the documentation of this file.

1 #ifndef STAN\_\_PROB\_\_DISTRIBUTIONS\_\_PARETO\_HPP

2 #define STAN\_\_PROB\_\_DISTRIBUTIONS\_\_PARETO\_HPP

3

4 #include <boost/random/exponential\_distribution.hpp>

5 #include <boost/random/variate\_generator.hpp>

6

7 #include <stan/agrad/partials\_vari.hpp>

8 #include <stan/math/error\_handling.hpp>

9 #include <stan/math/constants.hpp>

10 #include <stan/math/functions/multiply\_log.hpp>

11 #include <stan/math/functions/value\_of.hpp>

12 #include <stan/meta/traits.hpp>

13 #include <stan/prob/constants.hpp>

14 #include <stan/prob/traits.hpp>

15

16

17 namespace stan {

18  namespace prob {

19

20  // Pareto(y|y\_m,alpha) [y > y\_m; y\_m > 0; alpha > 0]

21  template <bool propto,

22  typename T\_y, typename T\_scale, typename T\_shape>

23  typename return\_type<T\_y,T\_scale,T\_shape>::type

24  pareto\_log(const T\_y& y, const T\_scale& y\_min, const T\_shape& alpha) {

25  static const char\* function = "stan::prob::pareto\_log(%1%)";

26

27  using stan::math::value\_of;

28  using stan::math::check\_positive\_finite;

29  using stan::math::check\_not\_nan;

30  using stan::math::check\_consistent\_sizes;

31

32  // check if any vectors are zero length

33  if (!(stan::length(y)

34  && stan::length(y\_min)

35  && stan::length(alpha)))

36  return 0.0;

37

38  // set up return value accumulator

39  double logp(0.0);

40

41  // validate args (here done over var, which should be OK)

42  check\_not\_nan(function, y, "Random variable", &logp);

43  check\_positive\_finite(function, y\_min, "Scale parameter", &logp);

44  check\_positive\_finite(function, alpha, "Shape parameter", &logp);

45  check\_consistent\_sizes(function,

46  y,y\_min,alpha,

47  "Random variable","Scale parameter",

48  "Shape parameter",

49  &logp);

50

51  // check if no variables are involved and prop-to

52  if (!include\_summand<propto,T\_y,T\_scale,T\_shape>::value)

53  return 0.0;

54

55  VectorView<const T\_y> y\_vec(y);

56  VectorView<const T\_scale> y\_min\_vec(y\_min);

57  VectorView<const T\_shape> alpha\_vec(alpha);

58  size\_t N = max\_size(y, y\_min, alpha);

59

60  for (size\_t n = 0; n < N; n++) {

61  if (y\_vec[n] < y\_min\_vec[n])

62  return LOG\_ZERO;

63  }

64

65  // set up template expressions wrapping scalars into vector views

66  agrad::OperandsAndPartials<T\_y,T\_scale,T\_shape>

67  operands\_and\_partials(y, y\_min, alpha);

68

69  DoubleVectorView<include\_summand<propto,T\_y,T\_shape>::value,

70  is\_vector<T\_y>::value> log\_y(length(y));

71  if (include\_summand<propto,T\_y,T\_shape>::value)

72  for (size\_t n = 0; n < length(y); n++)

73  log\_y[n] = log(value\_of(y\_vec[n]));

74

75  DoubleVectorView<!is\_constant\_struct<T\_y>::value

76  ||!is\_constant\_struct<T\_shape>::value,

77  is\_vector<T\_y>::value> inv\_y(length(y));

78  if (!is\_constant\_struct<T\_y>::value||!is\_constant\_struct<T\_shape>::value)

79  for (size\_t n = 0; n < length(y); n++)

80  inv\_y[n] = 1 / value\_of(y\_vec[n]);

81

82  DoubleVectorView<include\_summand<propto,T\_scale,T\_shape>::value,

83  is\_vector<T\_scale>::value>

84  log\_y\_min(length(y\_min));

85  if (include\_summand<propto,T\_scale,T\_shape>::value)

86  for (size\_t n = 0; n < length(y\_min); n++)

87  log\_y\_min[n] = log(value\_of(y\_min\_vec[n]));

88

89  DoubleVectorView<include\_summand<propto,T\_shape>::value,

90  is\_vector<T\_shape>::value> log\_alpha(length(alpha));

91  if (include\_summand<propto,T\_shape>::value)

92  for (size\_t n = 0; n < length(alpha); n++)

93  log\_alpha[n] = log(value\_of(alpha\_vec[n]));

94

95  using stan::math::multiply\_log;

96

97  for (size\_t n = 0; n < N; n++) {

98  const double alpha\_dbl = value\_of(alpha\_vec[n]);

99  // log probability

100  if (include\_summand<propto,T\_shape>::value)

101  logp += log\_alpha[n];

102  if (include\_summand<propto,T\_scale,T\_shape>::value)

103  logp += alpha\_dbl \* log\_y\_min[n];

104  if (include\_summand<propto,T\_y,T\_shape>::value)

105  logp -= alpha\_dbl \* log\_y[n] + log\_y[n];

106

107  // gradients

108  if (!is\_constant\_struct<T\_y>::value)

109  operands\_and\_partials.d\_x1[n] -= alpha\_dbl \* inv\_y[n] + inv\_y[n];

110  if (!is\_constant\_struct<T\_scale>::value)

111  operands\_and\_partials.d\_x2[n] += alpha\_dbl / value\_of(y\_min\_vec[n]);

112  if (!is\_constant\_struct<T\_shape>::value)

113  operands\_and\_partials.d\_x3[n]

114  += 1 / alpha\_dbl + log\_y\_min[n] - log\_y[n];

115  }

116  return operands\_and\_partials.to\_var(logp);

117  }

118

119  template <typename T\_y, typename T\_scale, typename T\_shape>

120  inline

121  typename return\_type<T\_y,T\_scale,T\_shape>::type

122  pareto\_log(const T\_y& y, const T\_scale& y\_min, const T\_shape& alpha) {

123  return pareto\_log<false>(y,y\_min,alpha);

124  }

125

126  template <typename T\_y, typename T\_scale, typename T\_shape>

127  typename return\_type<T\_y, T\_scale, T\_shape>::type

128  pareto\_cdf(const T\_y& y, const T\_scale& y\_min, const T\_shape& alpha) {

129

130  // Check sizes

131  // Size checks

132  if ( !( stan::length(y) && stan::length(y\_min) && stan::length(alpha) ) )

133  return 1.0;

134

135  // Check errors

136  static const char\* function = "stan::prob::pareto\_cdf(%1%)";

137

138  using stan::math::check\_positive\_finite;

139  using stan::math::check\_not\_nan;

140  using stan::math::check\_greater\_or\_equal;

141  using stan::math::check\_consistent\_sizes;

142  using stan::math::check\_nonnegative;

143  using stan::math::value\_of;

144

145  double P(1.0);

146

147  check\_not\_nan(function, y, "Random variable", &P);

148  check\_nonnegative(function, y, "Random variable", &P);

149  check\_positive\_finite(function, y\_min, "Scale parameter", &P);

150  check\_positive\_finite(function, alpha, "Shape parameter", &P);

151  check\_consistent\_sizes(function, y, y\_min, alpha,

152  "Random variable", "Scale parameter",

153  "Shape parameter", &P);

154

155  // Wrap arguments in vectors

156  VectorView<const T\_y> y\_vec(y);

157  VectorView<const T\_scale> y\_min\_vec(y\_min);

158  VectorView<const T\_shape> alpha\_vec(alpha);

159  size\_t N = max\_size(y, y\_min, alpha);

160

161  agrad::OperandsAndPartials<T\_y, T\_scale, T\_shape>

162  operands\_and\_partials(y, y\_min, alpha);

163

164  // Explicit return for extreme values

165  // The gradients are technically ill-defined, but treated as zero

166

167  for (size\_t i = 0; i < stan::length(y); i++) {

168  if (value\_of(y\_vec[i]) < value\_of(y\_min\_vec[i]))

169  return operands\_and\_partials.to\_var(0.0);

170  }

171

172  // Compute vectorized CDF and its gradients

173

174  for (size\_t n = 0; n < N; n++) {

175

176  // Explicit results for extreme values

177  // The gradients are technically ill-defined, but treated as zero

178  if (value\_of(y\_vec[n]) == std::numeric\_limits<double>::infinity()) {

179  continue;

180  }

181

182  // Pull out values

183  const double log\_dbl = log( value\_of(y\_min\_vec[n])

184  / value\_of(y\_vec[n]) );

185  const double y\_min\_inv\_dbl = 1.0 / value\_of(y\_min\_vec[n]);

186  const double alpha\_dbl = value\_of(alpha\_vec[n]);

187

188  // Compute

189  const double Pn = 1.0 - exp( alpha\_dbl \* log\_dbl );

190

191  P \*= Pn;

192

193  if (!is\_constant\_struct<T\_y>::value)

194  operands\_and\_partials.d\_x1[n]

195  += alpha\_dbl \* y\_min\_inv\_dbl \* exp( (alpha\_dbl + 1) \* log\_dbl )

196  / Pn;

197  if (!is\_constant\_struct<T\_scale>::value)

198  operands\_and\_partials.d\_x2[n]

199  += - alpha\_dbl \* y\_min\_inv\_dbl \* exp( alpha\_dbl \* log\_dbl ) / Pn;

200  if (!is\_constant\_struct<T\_shape>::value)

201  operands\_and\_partials.d\_x3[n]

202  += - exp( alpha\_dbl \* log\_dbl ) \* log\_dbl / Pn;

203  }

204

205  if (!is\_constant\_struct<T\_y>::value) {

206  for(size\_t n = 0; n < stan::length(y); ++n)

207  operands\_and\_partials.d\_x1[n] \*= P;

208  }

209  if (!is\_constant\_struct<T\_scale>::value) {

210  for(size\_t n = 0; n < stan::length(y\_min); ++n)

211  operands\_and\_partials.d\_x2[n] \*= P;

212  }

213  if (!is\_constant\_struct<T\_shape>::value) {

214  for(size\_t n = 0; n < stan::length(alpha); ++n)

215  operands\_and\_partials.d\_x3[n] \*= P;

216  }

217

218  return operands\_and\_partials.to\_var(P);

219  }

220

221  template <typename T\_y, typename T\_scale, typename T\_shape>

222  typename return\_type<T\_y, T\_scale, T\_shape>::type

223  pareto\_cdf\_log(const T\_y& y, const T\_scale& y\_min, const T\_shape& alpha) {

224

225  // Size checks

226  if ( !( stan::length(y) && stan::length(y\_min) && stan::length(alpha) ) )

227  return 0.0;

228

229  // Check errors

230  static const char\* function = "stan::prob::pareto\_cdf\_log(%1%)";

231

232  using stan::math::check\_positive\_finite;

233  using stan::math::check\_not\_nan;

234  using stan::math::check\_greater\_or\_equal;

235  using stan::math::check\_consistent\_sizes;

236  using stan::math::check\_nonnegative;

237  using stan::math::value\_of;

238

239  double P(0.0);

240

241  check\_not\_nan(function, y, "Random variable", &P);

242  check\_nonnegative(function, y, "Random variable", &P);

243  check\_positive\_finite(function, y\_min, "Scale parameter", &P);

244  check\_positive\_finite(function, alpha, "Shape parameter", &P);

245  check\_consistent\_sizes(function, y, y\_min, alpha,

246  "Random variable", "Scale parameter",

247  "Shape parameter", &P);

248

249  // Wrap arguments in vectors

250  VectorView<const T\_y> y\_vec(y);

251  VectorView<const T\_scale> y\_min\_vec(y\_min);

252  VectorView<const T\_shape> alpha\_vec(alpha);

253  size\_t N = max\_size(y, y\_min, alpha);

254

255  agrad::OperandsAndPartials<T\_y, T\_scale, T\_shape>

256  operands\_and\_partials(y, y\_min, alpha);

257

258  // Explicit return for extreme values

259  // The gradients are technically ill-defined, but treated as zero

260

261  for (size\_t i = 0; i < stan::length(y); i++) {

262  if (value\_of(y\_vec[i]) < value\_of(y\_min\_vec[i]))

263  return operands\_and\_partials.to\_var(stan::math::negative\_infinity());

264  }

265

266  // Compute vectorized cdf\_log and its gradients

267

268  for (size\_t n = 0; n < N; n++) {

269

270  // Explicit results for extreme values

271  // The gradients are technically ill-defined, but treated as zero

272  if (value\_of(y\_vec[n]) == std::numeric\_limits<double>::infinity()) {

273  return operands\_and\_partials.to\_var(0.0);

274  }

275

276  // Pull out values

277  const double log\_dbl = log( value\_of(y\_min\_vec[n])

278  / value\_of(y\_vec[n]) );

279  const double y\_min\_inv\_dbl = 1.0 / value\_of(y\_min\_vec[n]);

280  const double alpha\_dbl = value\_of(alpha\_vec[n]);

281

282  // Compute

283  const double Pn = 1.0 - exp(alpha\_dbl \* log\_dbl );

284

285  P += log(Pn);

286

287  if (!is\_constant\_struct<T\_y>::value)

288  operands\_and\_partials.d\_x1[n]

289  += alpha\_dbl \* y\_min\_inv\_dbl \* exp((alpha\_dbl + 1) \* log\_dbl) / Pn;

290  if (!is\_constant\_struct<T\_scale>::value)

291  operands\_and\_partials.d\_x2[n]

292  -= alpha\_dbl \* y\_min\_inv\_dbl \* exp( alpha\_dbl \* log\_dbl ) / Pn;

293  if (!is\_constant\_struct<T\_shape>::value)

294  operands\_and\_partials.d\_x3[n]

295  -= exp( alpha\_dbl \* log\_dbl ) \* log\_dbl / Pn;

296  }

297

298  return operands\_and\_partials.to\_var(P);

299  }

300

301  template <typename T\_y, typename T\_scale, typename T\_shape>

302  typename return\_type<T\_y, T\_scale, T\_shape>::type

303  pareto\_ccdf\_log(const T\_y& y, const T\_scale& y\_min,

304  const T\_shape& alpha) {

305

306  // Size checks

307  if ( !( stan::length(y) && stan::length(y\_min) && stan::length(alpha) ) )

308  return 0.0;

309

310  // Check errors

311  static const char\* function = "stan::prob::pareto\_ccdf\_log(%1%)";

312

313  using stan::math::check\_positive\_finite;

314  using stan::math::check\_not\_nan;

315  using stan::math::check\_greater\_or\_equal;

316  using stan::math::check\_consistent\_sizes;

317  using stan::math::check\_nonnegative;

318  using stan::math::value\_of;

319

320  double P(0.0);

321

322  check\_not\_nan(function, y, "Random variable", &P);

323  check\_nonnegative(function, y, "Random variable", &P);

324  check\_positive\_finite(function, y\_min, "Scale parameter", &P);

325  check\_positive\_finite(function, alpha, "Shape parameter", &P);

326  check\_consistent\_sizes(function, y, y\_min, alpha,

327  "Random variable", "Scale parameter",

328  "Shape parameter", &P);

329

330  // Wrap arguments in vectors

331  VectorView<const T\_y> y\_vec(y);

332  VectorView<const T\_scale> y\_min\_vec(y\_min);

333  VectorView<const T\_shape> alpha\_vec(alpha);

334  size\_t N = max\_size(y, y\_min, alpha);

335

336  agrad::OperandsAndPartials<T\_y, T\_scale, T\_shape>

337  operands\_and\_partials(y, y\_min, alpha);

338

339  // Explicit return for extreme values

340  // The gradients are technically ill-defined, but treated as zero

341

342  for (size\_t i = 0; i < stan::length(y); i++) {

343  if (value\_of(y\_vec[i]) < value\_of(y\_min\_vec[i]))

344  return operands\_and\_partials.to\_var(0.0);

345  }

346

347  // Compute vectorized cdf\_log and its gradients

348

349  for (size\_t n = 0; n < N; n++) {

350

351  // Explicit results for extreme values

352  // The gradients are technically ill-defined, but treated as zero

353  if (value\_of(y\_vec[n]) == std::numeric\_limits<double>::infinity()) {

354  return operands\_and\_partials.to\_var(stan::math::negative\_infinity());

355  }

356

357  // Pull out values

358  const double log\_dbl = log( value\_of(y\_min\_vec[n])

359  / value\_of(y\_vec[n]) );

360  const double y\_min\_inv\_dbl = 1.0 / value\_of(y\_min\_vec[n]);

361  const double alpha\_dbl = value\_of(alpha\_vec[n]);

362

363  P += alpha\_dbl \* log\_dbl;

364

365  if (!is\_constant\_struct<T\_y>::value)

366  operands\_and\_partials.d\_x1[n] -= alpha\_dbl \* y\_min\_inv\_dbl

367  \* exp(log\_dbl);

368  if (!is\_constant\_struct<T\_scale>::value)

369  operands\_and\_partials.d\_x2[n] += alpha\_dbl \* y\_min\_inv\_dbl;

370  if (!is\_constant\_struct<T\_shape>::value)

371  operands\_and\_partials.d\_x3[n] += log\_dbl;

372  }

373

374  return operands\_and\_partials.to\_var(P);

375  }

376

377  template <class RNG>

378  inline double

379  pareto\_rng(const double y\_min,

380  const double alpha,

381  RNG& rng) {

382  using boost::variate\_generator;

383  using boost::exponential\_distribution;

384

385  static const char\* function = "stan::prob::pareto\_rng(%1%)";

386

387  using stan::math::check\_positive\_finite;

388

389  check\_positive\_finite(function, y\_min, "Scale parameter", (double\*)0);

390  check\_positive\_finite(function, alpha, "Shape parameter", (double\*)0);

391

392  variate\_generator<RNG&, exponential\_distribution<> >

393  exp\_rng(rng, exponential\_distribution<>(alpha));

394  return y\_min \* std::exp(exp\_rng());

395  }

396  }

397 }

398 #endif

stan::prob::pareto\_rng

double pareto\_rng(const double y\_min, const double alpha, RNG &rng)

**Definition:** pareto.hpp:379

multiply\_log.hpp

traits.hpp

stan::agrad::OperandsAndPartials::to\_var

T\_return\_type to\_var(double logp)

**Definition:** partials\_vari.hpp:138

stan::math::check\_positive\_finite

bool check\_positive\_finite(const char \*function, const T\_y &y, const char \*name, T\_result \*result)

**Definition:** check\_positive\_finite.hpp:12

error\_handling.hpp

stan::math::multiply\_log

boost::math::tools::promote\_args< T\_a, T\_b >::type multiply\_log(const T\_a a, const T\_b b)

Calculated the value of the first argument times log of the second argument while behaving properly w...

**Definition:** multiply\_log.hpp:51

stan::length

size\_t length(const T &)

**Definition:** traits.hpp:159

stan::DoubleVectorView

DoubleVectorView allocates double values to be used as intermediate values.

**Definition:** traits.hpp:358

partials\_vari.hpp

stan::agrad::value\_of

T value\_of(const fvar< T > &v)

Return the value of the specified variable.

**Definition:** value\_of.hpp:16

constants.hpp

stan::is\_vector

**Definition:** traits.hpp:83

stan::agrad::OperandsAndPartials

A variable implementation that stores operands and derivatives with respect to the variable...

**Definition:** partials\_vari.hpp:76

traits.hpp

stan::return\_type::type

boost::math::tools::promote\_args< typename scalar\_type< T1 >::type, typename scalar\_type< T2 >::type, typename scalar\_type< T3 >::type, typename scalar\_type< T4 >::type, typename scalar\_type< T5 >::type, typename scalar\_type< T6 >::type >::type type

**Definition:** traits.hpp:406

stan::prob::pareto\_ccdf\_log

return\_type< T\_y, T\_scale, T\_shape >::type pareto\_ccdf\_log(const T\_y &y, const T\_scale &y\_min, const T\_shape &alpha)

**Definition:** pareto.hpp:303

stan::is\_constant\_struct

Metaprogram to determine if a type has a base scalar type that can be assigned to type double...

**Definition:** traits.hpp:57

stan::math::value\_of

double value\_of(const T x)

Return the value of the specified scalar argument converted to a double value.

**Definition:** value\_of.hpp:24

stan::prob::include\_summand

Template metaprogram to calculate whether a summand needs to be included in a proportional (log) prob...

**Definition:** traits.hpp:35

stan::agrad::OperandsAndPartials::d\_x2

VectorView< double \*, is\_vector< T2 >::value, is\_constant\_struct< T2 >::value > d\_x2

**Definition:** partials\_vari.hpp:83

stan::prob::pareto\_cdf\_log

return\_type< T\_y, T\_scale, T\_shape >::type pareto\_cdf\_log(const T\_y &y, const T\_scale &y\_min, const T\_shape &alpha)

**Definition:** pareto.hpp:223

stan::math::check\_nonnegative

bool check\_nonnegative(const char \*function, const T\_y &y, const char \*name, T\_result \*result)

**Definition:** check\_nonnegative.hpp:52

stan::math::check\_consistent\_sizes

bool check\_consistent\_sizes(const char \*function, const T1 &x1, const T2 &x2, const char \*name1, const char \*name2, T\_result \*result)

**Definition:** check\_consistent\_sizes.hpp:13

stan::max\_size

size\_t max\_size(const T1 &x1, const T2 &x2)

**Definition:** traits.hpp:191

stan::math::check\_not\_nan

bool check\_not\_nan(const char \*function, const T\_y &y, const char \*name, T\_result \*result)

Checks if the variable y is nan.

**Definition:** check\_not\_nan.hpp:56

value\_of.hpp

stan::agrad::OperandsAndPartials::d\_x1

VectorView< double \*, is\_vector< T1 >::value, is\_constant\_struct< T1 >::value > d\_x1

**Definition:** partials\_vari.hpp:82

stan::prob::pareto\_log

return\_type< T\_y, T\_scale, T\_shape >::type pareto\_log(const T\_y &y, const T\_scale &y\_min, const T\_shape &alpha)

**Definition:** pareto.hpp:24

stan::agrad::OperandsAndPartials::d\_x3

VectorView< double \*, is\_vector< T3 >::value, is\_constant\_struct< T3 >::value > d\_x3

**Definition:** partials\_vari.hpp:84

constants.hpp

stan::agrad::log

fvar< T > log(const fvar< T > &x)

**Definition:** log.hpp:15

stan::VectorView

VectorView is a template metaprogram that takes its argument and allows it to be used like a vector...

**Definition:** traits.hpp:275

stan::agrad::exp

fvar< T > exp(const fvar< T > &x)

**Definition:** exp.hpp:16

stan::math::check\_greater\_or\_equal

bool check\_greater\_or\_equal(const char \*function, const T\_y &y, const T\_low &low, const char \*name, T\_result \*result)

**Definition:** check\_greater\_or\_equal.hpp:58

stan::prob::pareto\_cdf

return\_type< T\_y, T\_scale, T\_shape >::type pareto\_cdf(const T\_y &y, const T\_scale &y\_min, const T\_shape &alpha)

**Definition:** pareto.hpp:128

stan::math::negative\_infinity

double negative\_infinity()

Return negative infinity.

**Definition:** constants.hpp:123


---

[ Stan Home Page ]

*© 2011–2014,
Stan Development Team.*
